# The Role of Social Functioning Between Vitality and Mental Distress in Hypertension: A Partial Mediation Model

**DOI:** 10.3390/ejihpe15050072

**Published:** 2025-05-07

**Authors:** Sara Guidotti, Francesca Giordano, Carlo Pruneti

**Affiliations:** 1 Clinical Psychology, Clinical Psychophysiology, and Clinical Neuropsychology Laboratories, Department of Medicine and Surgery, University of Parma, 43126 Parma, Italy; carlo.pruneti@unipr.it; 2 National Health Service of Lecce, 73100 Lecce, Italy; francesca_giord_23@libero.it

**Keywords:** hypertension, lifestyle, mediation analysis, quality of life, social support

## Abstract

(1) Background: Patients with hypertension face a relevant reduction in health-related quality of life. Specifically, the vitality domain is significantly impaired. This research aims to explore the association between quality of life and mental distress in patients with hypertension, explicitly emphasizing the mediating factor of social functioning above vitality. (2) Methods: This observational study consecutively recruited a hundred hypertensive patients (49% were males and 51% were females) aged between 23 and 82 years old (M_age_ = 56.04, SD_age_ = 12.04). The Symptom Checklist-90-Revised (SCL-90-R) and the 36-Item Short Form Health Survey (SF-36) were administered to assess mental distress and quality of life, respectively. Two biological measures (i.e., cortisol levels and heart rate) were also collected. (3) Results: In total, 50% of participants indicated higher mental distress and reduced quality of life. Correlation analyses demonstrate various negative relationships between clinical features. Moreover, positive associations were found between mental distress and vitality and between vitality and physical and social functioning along with heart rate. Notably, it was determined that vitality negatively predicted mental distress directly and indirectly by mediating social functioning. (4) Conclusions: Despite these promising findings, this study’s cross-sectional nature does not allow for the definition of the causal relationship between the investigated variables. These results emphasize the importance of a comprehensive and multidisciplinary evaluation in understanding hypertensive patients’ psychophysical well-being and lifestyles, which social support may significantly modulate.

## 1. Introduction

Hypertension is characterized by increased blood pressure (Li et al., 2018). Approximately 1 billion people worldwide suffer from hypertension, and this number is estimated to increase to 1.56 billion by 2025, involving 30% of the general population (World Health Organization, 2013). However, the actual prevalence is believed to be even higher due to the tendency to underestimate this condition (Carreira-Míguez et al., 2022).

Hypertension is recognized as one of the most significant modifiable risk factors for cardiovascular events (i.e., cardiac death, coronary heart disease, heart failure, and stroke) (Rosendorff et al., 2007). Since hypertension is the most prevalent risk factor for all forms of cardiovascular disease (GBD 2017 Disease and Injury Incidence and Prevalence Collaborators, 2017), it is crucial to identify its risk factors (Lee et al., 2019).

First, hypertension is influenced by numerous socio-demographic (i.e., family history, advanced age, etc.) and clinical factors (i.e., overweight, diabetes, etc.) (Hamam et al., 2020), as well as lifestyle (i.e., physical inactivity, tobacco use, etc.) (Riaz et al., 2021).

Furthermore, psychological comorbidity has been frequently documented (Bai et al., 2021). Recent studies describe a coexistence of anxiety and depression in patients with arterial hypertension (Hryn et al., 2021; Polishchuk et al., 2021; Villarreal-Zegarra & Bernabe-Ortiz, 2020). Anxiety, being supported by excessive activity of the autonomic nervous system and the hypothalamic–pituitary–adrenal axis, could also be the cause of an increase in blood pressure (Lambert et al., 2010). To illustrate, the results of the 2005 US National Ambulatory Medical Care Survey and the National Hospital Ambulatory Medical Care Survey indicated that nearly 32.5% of outpatients seeking treatment for anxiety had undiagnosed hypertension (Player et al., 2008).

Conversely, individuals with chronic medical conditions (i.e., hypertension) may experience negative emotions that contribute to the production of anxious and depressive manifestations (Dudek et al., 2021; Ivanušić Pejić & Degmečić, 2022). Additionally, high rates of psychiatric comorbidities, such as anxiety and depression, significantly reduce the quality of life for these patients (Ivanušić Pejić & Degmečić, 2022; Polishchuk et al., 2021). It should also be noted that psychological factors are associated with a reduced likelihood of adhering to prescribed medical treatments (Hedegaard et al., 2015; Kretchy et al., 2014). This complex interaction may lead to treatment-resistant hypertension, medication non-adherence (including both medication intake and adherence to lifestyle modifications), and, ultimately, worse health outcomes (Colivicchi et al., 2010; Souza et al., 2016).

Focusing on non-pharmacological prescriptions and lifestyle, Li et al. (2018) observed that healthy behaviors were positively correlated with perceived quality of life in hypertensive patients (Acton & Malathum, 2000; Holahan & Suzuki, 2004). The researchers suggested that hypertension, as a behavior-related disease, is closely linked to unhealthy lifestyle choices. Factors such as physical inactivity, the higher prevalence of mental and physical disorders, nursing shortages, low-income, and social isolation often contribute to feelings of loneliness and depression. Such challenges may, in turn, reduce the likelihood of engaging in health-promoting behaviors (Li et al., 2018). Furthermore, the same authors highlighted a moderate decline in health-related quality of life, particularly in the role emotional and vitality domains, which consistently scored below average. Carreira-Míguez et al. (2022) also explored eating habits and perceived vitality during weekdays and weekends, finding that hypertensive patients exhibited unhealthy eating patterns, favoring lower vitality levels during the week. Although recent research already examined vitality, this aspect has been predominantly studied in regard to lifestyle factors, such as nutrition and physical activity (Arija et al., 2018).

To our knowledge, the connection between mental distress and reduced vitality in hypertensive patients has not been extensively explored. This research aims to address this gap by investigating how the aspect of vitality interacts with mental distress in individuals suffering from hypertension. In particular, this study will examine whether certain interpersonal factors, such as social functioning, might influence the relationship between vitality and psychological distress.

Thus, the following hypotheses were tested: (1) hypertensive patients with mental distress report worse autonomic arousal (i.e., cortisol levels and heart rate) and quality of life compared to a non-clinical group of hypertensive patients; (2) vitality as a significant predictor of mental distress; and (3) social functioning acting as a mediator in the relationship between vitality and mental distress.

**Figure 1:**
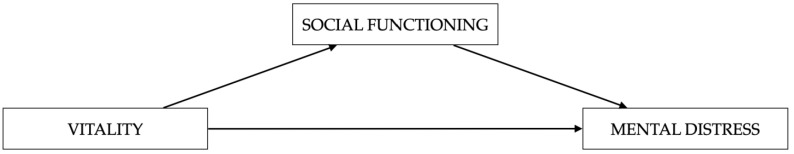
The mediation model explored.

The tested mediation model is shown in Figure 1.

## 2. Materials and Methods

### 2.1. Participants and Procedure

The study design was observational and case–control.

This research consecutively recruited a total of one hundred individuals with arterial hypertension. Patients who visited the Cardiac Rehabilitation Service at the San Cesario Hospital in Lecce (Southern Italy) and who received a medical diagnosis of arterial hypertension were enrolled.

Inclusion criteria were the following: (1) adult age; (2) confirmed diagnosis of arterial hypertension; (3) absence of physical or mental illnesses that could have hindered the administration of psychological questionnaires; and the (4) absence of pharmacological treatments in progress at the time of the visit.

The project took place within the San Cesario Hospital in Lecce, which provided a room to administer the psychological questionnaires in a 60 min in-person appointment. A PhD student in Clinical Psychology was responsible for administering the questionnaires and collecting medical data (i.e., heart rate and cortisol) from the patient’s medical records between January and December 2018.

Heart rate was taken at the same time as blood pressure measurement, performed using a pulse oximeter. Cortisol value was collected by taking a blood sample.

The experimental procedures were completed with the 1964 Declaration of Helsinki of the World Medical Association and the 2005 Universal Declaration on Bioethics and Human Rights of UNESCO.

### 2.2. Measures

The following standardized questionnaires were administered to participants.

The Symptom Checklist-90-Revised (SCL-90-R; Derogatis, 1994; Prunas et al., 2012) is a standardized questionnaire for measuring psychological symptoms and their severity. The SCL-90-R is composed of 90 items with Likert responses from 1 to 5. The participant is asked to respond by referring to the internalizing and externalizing manifestations experienced in the last seven days. The clinical scales are the following: somatization (SOM; 12 items), obsessive-compulsive (O-C; 10 items), interpersonal sensitivity (I-S; 9 items), depression (DEP; 13 items), anxiety (ANX; 10 items), hostility (HOS; 6 items), phobic anxiety (PHOB; 7 items), paranoid ideation (PAR; 6 items), and psychoticism (PSY; 10 items). The Global Severity Index (GSI) (α = 0.87) is an indicator of the depth of mental distress experienced by the individual, relating the number of reported symptoms to the intensity of perceived distress. The raw score of each scale is converted into a T-point scale, where T scores equal to or greater than 63 in two or more scales or the GSI scale indicates the presence of a clinically significant psychological problem (Derogatis & Svitz, 1999). The symptom dimensions have acceptable to excellent Cronbach’s alpha, ranging from 0.67 (PHOB) to 0.87 (DEP).

The Short Form Health Survey (SF-36; Apolone & Mosconi, 1998) is a standardized questionnaire used to investigate perceived quality of life. The SF-36 is composed of 36 items grouped into 8 domains: physical functioning (PF; 10 items), role physical (RF; 4 items), bodily pain (BP; 2 items), general health (GH; 5 items), vitality (VT; 4 items), social functioning (SF; 2 items), role emotional (RE; 3 items), and mental health (MH; 5 items). The domains are conveyed into two main scales, the Physical Component Synthesis (PCS) and the Mental Component Synthesis (MCS), which globally represent physical and mental health, respectively. Higher scores in these scales reflect better health-related quality of life (HRQoL). Cronbach’s alpha values range from 0.64 to 0.71.

### 2.3. Statistical Analysis

All statistical analyses were carried out using SPSS (Version 28.0.1.0; IBM Corp., Armonk, NY, USA). The differences in socio-demographic (i.e., age, gender, marital status, educational level, and current occupational) and clinical features (i.e., mental distress, quality of life, and medical data, including cortisol and heart rate) between the clinical group (i.e., individuals with scores exceeding the cut-off value of 63 T points on the GSI scale of the SCL-90-R) and the non-clinical group were analyzed using Chi-squared tests or independent samples *t*-tests.

A Pearson’s correlation analysis was conducted to explore the relationships between the clinical features (i.e., mental distress, quality of life, cortisol, and heart rate). Consequently, a serial mediation analysis was implemented using the PROCESS macro for SPSS v22 (Hayes, 2017). Model 4 of the PROCESS macro was applied to include vitality (assessed through SF-26 VT) as the independent variable, social functioning (assessed through SF-36 SF) as the serial mediator, and mental distress (assessed through SCL-90-R GSI) as the dependent variable.

## 3. Results

### 3.1. Sample Characteristics and Comparison Between Clinical and Non-Clinical Patients

A standardized Cohen’s effect size of 0.15 was utilized in this study, along with a type I error rate of 5% (α = 0.05) and a type II error rate of 5% (β = 0.05; power = 95%). An a priori power analysis conducted using GPower 3.1 determined that a sample size of 90 participants was necessary (Fritz & Mackinnon, 2007). Taking into account a drop-out rate of 10%, a sample of 100 people was formed. Since no drop-outs were verified, the post hoc power analysis indicated that the achieved power of the actual sample was equal to 0.97.

According to the GSI score, a clinical group (GSI score ≥ 63) composed of 50 people was separated from a non-clinical one (GSI score < 63) comprising 50 people.

A description of the socio-demographic features is shown in Table 1.

The age of participants ranged from 23 to 82 years, with a mean age of 56.04 ± 12.04 years. The sample was evenly distributed by gender, comprising 49% males and 51% females. Most participants held middle (60%) or high school diplomas (30%), while the majority were either employed (56%) or retired (26%). Regarding marital status, 86% of participants were married or cohabiting.

However, it should be noted that the clinical sample was mostly composed of women and characterized by lower education than the non-clinical group.

A description of the clinical features is shown in Table 2.

Looking at the mental distress of the total sample of hypertensive patients, no scale of the SCL-90-R exceeded the cut-off of 63 T points. Moderately elevated scores can be noted for the somatization scale, obsessions-compulsions, depression, and anxiety. In contrast, quality of life is more compromised, with one standard deviation (15 percentile points) exceedance for physical and social functioning. The scales of bodily pain, general health, vitality, and mental health exceeded two standard deviations (30 percentile points).

A comparison between the two groups of patients reveals that mental distress is significantly higher in the clinical group. Specifically, all clinical scales of the SCL-90-R are higher in the clinical group than in the non-clinical group, exceeding the cut-off of 63 T points for obsessions-compulsions, depression, and anxiety and two standard deviations for somatizations.

The clinical group reported a decline in quality of life that was also significantly greater than the non-clinical group, with lower values in all scales except general health.

A significant difference also emerged concerning the value of cortisol, although it is at the limits of statistical significance.

### 3.2. Correlations Between the Clinical Variables

The relationships among the variables are detailed in Table 3.

The GSI scale of the SCL-90-R negatively correlated with the SF-36 scales that evaluate physical and social functioning, general and mental health, and vitality.

Furthermore, vitality was associated with medical data (i.e., heart rate) and social and physical functioning, as well as mental health.

### 3.3. Serial Mediation Analysis

**Figure 2:**
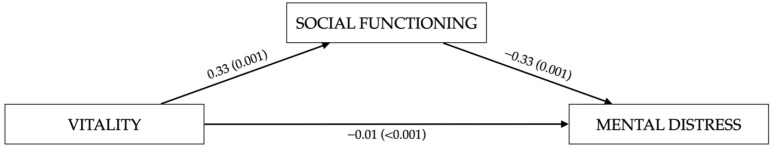
Results of the partial mediation model (β, *p*). All coefficients are standardized.

The mediation model is presented in Figure 2. We tested a model in which vitality (SF-26 V) was hypothesized to be a significant predictor of mental distress (SCL-90-R GSI) directly and indirectly through the involvement of social functioning (SF-36 SF). The model revealed that vitality was significantly associated with mental distress [B = −0.01; SE = 0.002; *p* = 0.001; LLCI-ULCI (−0.01, −0.003)] as well as social functioning [B = 0.53; SE = 0.10; *p* < 0.001; LLCI-ULCI (0.33, 0.72)], which was a significant predictor of mental distress [B = −0.01; SE = 0.002; *p* = 0.001; LLCI-ULCI (−0.01, −0.003)]. Furthermore, the mediation analysis certified that social functioning partially mediated the relationship between vitality and mental distress [B = −0.16; SE = 0.05; *p* = 0.001; LLCI-ULCI (−0.26, −0.06)]. The model including gender (coded as 1 = female and 0 = male) [B = −0.05; SE = 0.08; *p* = 0.11; LLCI-ULCI (−0.21, 0.11)] and age [B = −0.01; SE = 0.003; *p* = 0.001; LLCI-ULCI (− 0.01, 0.001)] as covariates explained 59% of the variance (F (4, 95) = 12.50; *p* < 0.001).

## 4. Discussion

The analysis of mental distress in the total sample did not reveal characteristics worthy of clinical interest, except for moderate scores for the somatization, obsessions-compulsions, depression, and anxiety scales. However, when dividing the group according to mental distress, the same clinical scales of the SCL-90-R were higher in the clinical group of hypertensive patients, indicating the presence of psychological symptoms relevant to their mental health.

On the contrary, the decline in perceived quality of life was more evident on all scales, even in the total sample, with scores significantly lower than 100 by one or two standard deviations. Also in this case, the clinical group reported significantly lower scores than the non-clinical group, except for the general health scale. In other words, when asked to reflect on personal and social functioning, hypertensive patients showed a greater perception of suffering.

Thus, the comparison between the two groups highlighted the clinical characteristics of interest, consistent with previous studies that calculated a high prevalence of psychiatric comorbidities (Ivanušić Pejić & Degmečić, 2022; Kupper & Denollet, 2018; Oliva et al., 2016; Polishchuk et al., 2021). As already noted, patients in the clinical sample reported relevant psychological symptoms along with a worse quality of life. Specifically considering the SF-36 scales, vitality received the lowest score, corroborating previous research (Carreira-Míguez et al., 2022; Li et al., 2018). This aspect has been revisited only recently, although it was already investigated by Levine et al. in 1987. The authors suggested the importance of paying attention to the perception of vitality of this category of patients, raising the issue of the side effects (i.e., fatigue and sleepiness) of antihypertensive drugs. More recently, a review of the literature by Souza et al. (2016) appeared to be more reassuring, documenting conflicting results with both the negative and positive effects of pharmacological and non-pharmacological therapies (i.e., specific programs for adapted physical activity). In summary, it is a widespread belief that recent pharmacological advances can significantly improve the quality of life of the hypertensive patient, both for his general and mental health, emphasizing the importance of early diagnosis for timely treatment (Arija et al., 2018).

The investigation of associations between variables also attested to several clinical aspects worthy of note. First, among the biological parameters assessed, a moderate association was found between heart rate and physical functioning, along with a slight positive correlation with vitality. In other words, higher levels of vitality and physical functioning were associated with an increase in heart rate. This finding is interesting, but worrying, especially in a group of hypertensive patients, for whom a primary health goal is to maintain controlled cardiac activity. Therefore, it is fundamental to formulate specific hypotheses. One possibility is that hypertensive individuals tend to be hyper-aroused both at the psychophysiological and behavioral levels, where a decrease in their activity levels can generate psychological distress. If this is the case, it underlines the functional limitations caused by physical diseases. It is not uncommon for lively, vital, energetic, and hyper-aroused individuals to consider medical recommendations as limitations reducing their activity levels (Schiavoni et al., 2023; Villarreal-Zegarra & Bernabe-Ortiz, 2020). Another hypothesis is that hypertensive patients are more likely to feel more active and energetic when their perceived well-being aligns with increased autonomic reactivity. This aspect, in turn, may contribute to elevated blood pressure levels and an increased risk for their physical health (Bonaguidi et al., 1996; Rosenman, 1991). However, this aspect should be explored using appropriate tools designed to detect specific personality traits, such as standardized questionnaires and psychometric tests, as researchers made with post-myocardial infarction and heart failure patients (Wirtz & von Känel, 2017).

The analysis of correlations between clinical measures revealed a strong relationship between quality of life and mental distress reported by the GSI of the SCL-90-R. In particular, one of the most significant correlations was found between vitality and mental distress. Vitality emerged as a notable factor in the perception of one’s health status, suggesting that a decrease in perceived energy levels corresponds to a worsening of mental health. Within this relationship, social functioning was described as a significant mediating factor. However, it is important to emphasize that vitality remains a significant predictor of mental distress even after including social functioning as a mediator, indicating that the perception of energy and vigor remains a key point for the mental health of these patients. The mediation model was partial, highlighting the role of social functioning in influencing only a part of the relationship between quality of life and psychological symptoms.

Notwithstanding, the significance of the serial mediation analysis is in line with the literature exploring the psychological dimensions involved in psychosomatic mechanisms (Hryn et al., 2021; Ivanušić Pejić & Degmečić, 2022; Polishchuk et al., 2021; Villarreal-Zegarra & Bernabe-Ortiz, 2020). The neuroscientific literature indicates that, in addition to factors that trigger sympathetic drive, such as mental distress and low quality of life, there are also elements that stimulate the parasympathetic nervous system, which counteracts sympathetic activity (Porges, 2009). In line with the Polyvagal Theory developed by Porges, social support is recognized as a vital factor that promotes a harmonious balance between the sympathetic and parasympathetic branches of the autonomic nervous system (Porges, 1995, 2003). In agreement with the scientific evidence, the social engagement system activates calming circuits associated with parasympathetic activity, thus facilitating social interactions that help attenuate defensive responses associated with fight-or-flight reactions (Porges, 2021).

To our knowledge, social functioning has been identified as a mediating factor between vitality and mental distress in hypertensive patients for the first time. Our results support the experimental hypothesis, indicating that the connection between vitality and mental health is mediated by engagement in social activities. Mediation analysis seems to demonstrate that increased energy levels impact mental distress, both directly and indirectly, by favoring participation in social interactions. In summary, social engagement allows for hypertensive patients to improve their mental health, counteracting mental distress. In other words, higher levels of vitality encourage engagement in social activities, which, in turn, helps reduce perceived distress. Conversely, lower energy levels may interfere with interpersonal functioning, decreasing participation in social activities that are useful to protect mental health.

The promising findings of our research must be read in light of the current limitations. Although the presence of socio-demographic factors (i.e., gender and educational level) is consistent with the literature (Hamam et al., 2020), the lack of control of educational level (which was at the limit of significance) represents a non-negligible limitation. It would be appropriate for future research to control all of the significant covariates. Nonetheless, our observational and cross-sectional study does not allow us to better define the causal nature of the variables investigated. This highlights the need for further research to clarify the relationship between a decline in vitality and the worsening of mental distress. It would be helpful to define the predisposing, precipitating, and chronic impact of psychological factors on physical health. In this regard, it could also be interesting to examine whether social functioning has a positive influence on the cardiac activity of these patients.

If confirmed, these results could indicate that a significant portion of hypertensive patients could have an etiology for their condition related to psychological factors that predispose them to stress-related physical disorders. As repeatedly observed in the literature, autonomic hyperarousal could act as a link between personality/lifestyle and cardiac dysfunction, as psychophysical stress could contribute to the development or acceleration of severe cardiac accidents (Pruneti et al., 2002).

In light of all these assumptions, investing energy in this direction seems to be crucial for hypertensive patients and their families, as well as the national health system.

A clinical psychological assessment that uses standardized tools is essential to intercept mental suffering among hypertensive patients. However, it should not be overlooked that, in the hypertensive population, psychological symptoms are not as evident as deteriorations in quality of life. The analysis of personal and interpersonal functioning should, therefore, not be absent in clinical contexts that deal with hypertension. Communication between mental health professionals and cardiologists could also be facilitated by the use of specific measures, easy to understand and share, such as some medical data relating to autonomic arousal (i.e., cortisol, heart rate).

Furthermore, investigating social functioning could highlight a useful factor in improving the perception of vitality and mental well-being. On the contrary, greater involvement in social contexts could be an insightful suggestion to offer in psychoeducational and counseling programs, given its ability to mediate the effect of vitality on mental distress. Promoting healthy lifestyles and behaviors functional to improving the mental health of patients with hypertension should be at the center of the policies of the national health system. The support of non-pharmacological strategies for the management of patients in cardiac services for hypertension could reduce the progressive impoverishment of their physical and mental health and, consequently, the number of visits and admissions to hospital departments, favoring the better control of costs and public resources.

## 5. Conclusions

Our findings illuminate an underexplored medical condition and emphasize the necessity of investigating the psychological factors associated with hypertension. It is especially fundamental to take into account lifestyle choices and individuals’ perceived levels of vitality and energy, as even a slight decline in quality of life can be perceived by hypertensive patients as a significant limitation. While this aspect has drawn researchers’ attention, both the quality of life and the perception of vitality remain prominent yet insufficiently explored features in clinical practice. Furthermore, these psychological processes may influence the psychobiological mechanisms that contribute to the development of hypertension. Importantly, a heightened sense of vitality can enhance participation in social activities, which may positively impact the psychopathological symptoms experienced by these individuals. In summary, despite certain limitations, this study is one of the first to explore the mediating role of social functioning in the relationship between quality of life, including vitality, and mental health in individuals with hypertension. Given this evidence, future research should aim to determine whether this factor can serve as a non-pharmacological approach to managing the condition. From a clinical standpoint, medical teams should integrate a psychological evaluation that assesses emotional experiences, quality of life, and the influence of social interactions on alleviating suffering.

## Figures and Tables

**Table 1:** Comparisons of socio-demographic features between the non-clinical group and the clinical group of hypertensive patients.

Variable		Non-Clinical Group (n = 50)	Clinical Group (n = 50)	Total Sample (n = 100)	t or χ^2^	*p*
Age, M (SD)		55 (11.4)	57.1 (12.7)	56.04 (12.04)	t (98) = −0.88	0.48
Sex, N (%)					χ^2^ (1, n = 100) = 6.79	0.01
	Male	32 (32%)	17 (17%)	49 (49%)		
	Female	19 (19%)	32 (32%)	51 (51%)		
Marital status, N (%)					χ^2^ (3, n = 100) = 4.81	0.18
	Married/cohabitating	44 (44%)	42 (42%)	86 (86%)		
	Unmarried	4 (4%)	1 (1%)	5 (5%)		
	Separated/divorced	1 (1%)	5 (5%)	6 (6%)		
	Widowed	2 (2%)	1 (1%)	3 (3%)		
Education Level, N (%)					χ^2^ (2, n = 100) = 5.36	0.07
	Middle school graduation	25 (25%)	35 (35%)	60 (60%)		
	High school graduation	20 (20%)	10 (10%)	30 (30%)		
	University degree	6 (6%)	4 (4%)	10 (10%)		
Current Occupation, N (%)					χ^2^ (3, n = 100) = 1.24	0.74
	Student	0 (0%)	1 (1%)	1 (1%)		
	Employed	29 (29%)	27 (27%)	56 (56%)		
	Not employed	8 (8%)	9 (9%)	17 (17%)		
	Retired	14 (14%)	12 (12%)	26 (26%)		

**Table 2:** Comparisons of clinical features between the non-clinical group and the clinical group of hypertensive patients.

		Non-Clinical Group (n = 50)		Clinical Group (n = 50)		Total Sample (n = 100)		t (98)	*p*
		M	SD	M	SD	M	SD		
Symptom Checklist-90-Revised									
	Somatization	52.28	8.61	70.72	18.01	61.32	16.79	−7.54	<0.001
	Obsessive-Compulsive	48.35	7.28	64.73	14.31	56.38	13.92	−8.11	<0.001
	Interpersonal sensitivity	46.38	4.98	55.85	12.64	51.02	10.61	−5.11	<0.001
	Depression	49.77	8.78	64.40	15.92	56.94	14.69	−7.13	<0.001
	Anxiety	50.21	8.07	66.66	16.42	58.27	15.23	−7.41	<0.001
	Hostility	47.58	6.99	55.01	10.01	51.22	9.34	−5.81	<0.001
	Phobic anxiety	47.84	3.87	58.29	18.75	52.96	14.34	−3.93	<0.001
	Paranoid ideation	47.63	6.09	60.83	16.21	54.10	13.78	−5.92	<0.001
	Psychoticism	47.77	5.08	62.28	17.19	54.88	14.47	−6.08	<0.001
36-item Short Form Health Survey									
	Physical functioning	90.88	12.80	77.65	19.64	84.40	17.71	4.36	<0.001
	Bodily pain	79.88	23.90	58.20	27.12	69.26	27.63	4.61	<0.001
	General health	55.06	11.46	54.71	12.67	54.89	12.01	0.26	0.28
	Vitality	69.61	17.97	50.51	21.70	60.25	21.51	5.89	<0.001
	Social functioning	81.18	13.89	60.51	26.80	71.05	23.52	5.00	<0.001
	Mental health	76.00	16.04	56.82	21.73	66.60	21.25	5.97	<0.001
Medical Measurements									
	Cortisol (in μg/dL)	14.42	4.76	15.76	4.19	15.05	4.52	−1.32	0.050
	Heart Rate (in bpm)	69.17	8.47	69.73	9.86	69.43	14.47	−0.29	0.39

**Table 3:** Relationships between clinical features.

	M	SD	1	2	3	4	5	6	7	8
1. Cortisol	15.05	4.52	-							
2. Heart Rate	69.43	9.09	-							
3. SCL-90-R GSI	0.54	0.46	-	-						
4. SF-36 PF	84.40	17.71	-	0.24 *	−0.33 **					
5. SF-36 BP	69.26	27.63	-	-	-	-				
6. SF-36 GH	54.12	12.01	-	-	−0.19 *	-	-			
7. SF-36 VT	60.25	21.51	-	0.19 *	−0.51 *	0.53 **	-	-		
8. SF-36 SF	71.05	23.52	-	-	−0.48 **	0.53 **	-	-	0.50 **	
9. SF-36 MH	66.60	21.25	-	-	−0.66 **	0.40 **	-	0.72 **	0.59 **	-

Legend: * = *p* < 0.05, ** = *p* < 0.01; SCL-90-R = Symptom Checklist-90-Revised; GSI = Global Severity Index; SF-36 = Short Form Health Survey-36; PF = physical functioning; BP = bodily pain; GH = general health; VT = vitality; SF = social functioning; MH = mental health.

## Data Availability

The data presented in this study are available upon reasonable request from the corresponding author.
